# The interactive effect of affectionate nicknames and streamer type on streamer attitude in E-commerce live streaming: the mediating role of psychological closeness

**DOI:** 10.3389/fpsyg.2024.1498235

**Published:** 2024-12-24

**Authors:** Yuan Liu, Maohong Liu

**Affiliations:** ^1^School of Economics and Management, Wuhan University, Wuhan, China; ^2^Management School, Wuhan University of Science and Technology, Wuhan, China

**Keywords:** streamer types, affectionate nicknames, psychological closeness, streamer attitude, virtual streamer, live streaming commerce

## Abstract

**Background:**

With the widespread application of Artificial Intelligence technology in the field of E-commerce, human–machine relationships have attracted considerable attention within the field of psychology. Address forms, as crucial linguistic cues, have shown notable progress in advancing research on interpersonal relationships; however, a comprehensive understanding of the dynamics in interpersonal (or human–machine) relationships among interactors remains elusive. Therefore, based on Social Identity Theory, this paper explores the interactive effects and underlying mechanisms of affectionate nicknames and streamer type on streamer attitude in E-commerce live streaming, with consumers’ perceptions of psychological closeness serving as the mediating mechanism.

**Methods:**

Two between-subjects experimental designs, both involving a 2 (Affectionate Nicknames: use vs. non-use) × 2 (Streamer Type: human streamer vs. virtual streamer) factorial structure, were conducted to test the hypotheses. Study 1, utilizing image materials, collected 368 samples on the *Credamo* to examine the interactive influence of streamer type and affectionate nicknames on streamer attitude. Study 2, employing video materials, gathered 370 samples on the same platform. While replicating and validating the findings of Study 1, it also revealed that the interactive effect of streamer type and affectionate nicknames on consumer’s streamer attitude is mediated by psychological closeness.

**Results:**

This paper finds that consumers’ psychological closeness differs significantly across different streamer types, leading to potential variations in consumer attitude toward streamer type within different linguistic communication contexts. Specifically, when consumers encounter human streamers, using affectionate nicknames elicits a more positive streamer attitude compared to not using them; conversely, when consumers encounter virtual streamers, not using affectionate nicknames results in a more negative streamer attitude compared to using them.

**Conclusion:**

This paper not only compares the linguistic and communicative distinctions between interpersonal relationships and human–machine relationships from a psychological perspective but also undertakes valuable empirical investigations into their interaction differences. Furthermore, it contributes to advancing research into consumer responses to commercial avatars and offers practical managerial guidance for businesses employing avatars in E-commerce live streaming practices.

## 1 Introduction

In recent years, with the rapid development of live streaming technology, E-commerce live streaming has emerged as a significant form of consumer shopping experience (Luo X. et al., 2024; Luo L. et al., 2024). Concurrently, advancements in artificial intelligence technology have led to the widespread adoption of virtual streamers by businesses, owing to their advantages, such as cost-efficiency, high productivity, and 24/7 availability. However, despite facing vast market opportunities, virtual streamers also encounter challenges, including a lack of social presence and brief consumer interaction durations (Gao et al., 2024). To enhance interactivity and create a more engaging ambiance in live streaming sessions, virtual streamers have begun mimicking the language and behavior of human streamers, particularly by employing affectionate nicknames to foster emotional bonds with consumers (Leech, 2014; Wang, 2022; Cheng, 2022). Despite these efforts to mimic human interaction styles, whether the effects of affectionate nicknames in human–machine relationships are analogous to those in interpersonal relationships remains a critical issue for further exploration. This issue not only holds significant practical implications for guiding the communication strategies of virtual streamers but also enhances our understanding of human–machine relationships between consumers and service robots.

In the rapidly emerging field of E-commerce live streaming, virtual streamers, as representatives of service-oriented avatars, utilize their AI-driven technological advantages to deliver consumers with unparalleled live streaming service experiences. The academic community has paid significant attention to this phenomenon, engaging in extensive discussions within the three-dimensional framework of “Streamers-Products-Scenes” (Wu et al., 2020), with the aim of uncovering how virtual streamers positively influence consumer cognition, attitudes, and behaviors (Bailenson and Cummings, 2006). In the “Streamers” dimension, research has demonstrated that enhancing the anthropomorphic characteristics and human-like language communication abilities of virtual streamers can substantially increase consumer acceptance and purchase intention. For example, Xu et al. (2024) explored the human-like personality of virtual streamers and found that it positively influences parasocial interactions, which further affect brand image. Gao et al. (2025) verified, based on the Stereotype Content Model (SCM), that the coolness factors of virtual streamers can also stimulate consumer purchasing desires. Additionally, Gong and Sun (2025) further pointed out that compared to rational language, the use of emotional language by virtual streamers is more likely to make consumers follow their advice. In the “Products” dimension, research has concentrated on the alignment between virtual streamers and various types of products. Yan et al. (2024) found that although virtual streamers are marginally less effective than human streamers in enhancing consumers’ purchase intention for hedonic products, they still exhibit substantial potential. Yao et al. (2024), using the SCM model, revealed the unique impact of virtual streamers’ use of socially oriented language on consumers’ purchase intention for experiential products. Regarding the “Scenes” dimension, Shao (2024) conducted an in-depth analysis of how consumer motivation, personality differences, and innovation barriers intertwine to jointly influence consumers’ intention to switch to virtual streamers, considering situational causation, multifocality, equifinality, and causal asymmetry. However, despite the rich findings within the “Streamers-Products-Scenes” framework, a critical and subtle linguistic cue—the initial address forms in interpersonal communication—has been largely overlooked in the exploration of the “Streamers” dimension. Address forms, as the initial remarks in interpersonal communication, not only reveal the speaker’s self-perception and positioning of the interaction partner’s relationship but also subtly delineate the common attributes and group boundaries between the two parties (Levinson, 2001; Clyne et al., 2009). In non-kinship interactions, the use of kinship terms serves as an effective strategy to reduce interpersonal distances and enhance intimacy (Leech, 2014). Yet, in the context of human–machine relationships, the effects of address forms remain underexplored, representing a critical gap in the existing literature. To address this gap, this study will examine the differential effects of affectionate nicknames in interpersonal versus human–machine relationships, with a particular focus on how streamer type (human streamer vs. virtual streamer) and affectionate nicknames jointly influence consumer attitudes toward streamers in E-commerce live streaming. Through a comparative analysis, we seek to uncover the distinct mechanisms of affectionate nicknames in human–machine interactions, thereby offering novel perspectives and insights for the future development of E-commerce live streaming.

Thus, grounded in Social Identity Theory, this paper explores the interaction effects between anchor types (human anchors vs. virtual anchors) and the use of intimate language (with or without) in E-commerce live streaming, uncovering the underlying mechanisms and environmental factors. Through two experimental designs, we find that both anchor type and the use of intimate language significantly interact to influence attitudes toward the anchor, with psychological closeness playing a critical mediating role in this process. This paper not only compares the differences between interpersonal and human–machine language communication from a psychological perspective and empirically explores their distinct interactions, enriching research on consumer responses to commercial virtual avatars, but also provides valuable managerial insights for the application of virtual streamers in E-commerce live streaming, offering practical significance for advancing the field of human–machine interaction.

## 2 Literature review

### 2.1 Interactivity of E-commerce live streamers

With the emergence of the “Live streaming for all” phenomenon in China’s online live streaming market, the number of E-commerce live streaming users has approached 500 million, making it a prominent mode for consumers to shop and unwind. Meanwhile, advancements in artificial intelligence have facilitated the widespread adoption of virtual avatars in the E-commerce sector, endowing them with transactional and social interaction capabilities comparable to those of human streamers (Foster et al., 2022). This shift has sparked considerable academic interest in the interactivity of E-commerce live streamers, covering various aspects, such as viewer engagement (Zhang et al., 2024), purchase intention (Liao et al., 2023; He et al., 2024), and the influence of streamer expertise on interaction effectiveness (Luo X. et al., 2024; Luo L. et al., 2024). For example, Zhang et al. (2024) found that a task-oriented interaction style adopted by streamers increases consumers’ cognitive vigilance, subsequently decreasing their engagement. Grounded in SOR theory, Tong et al. (2024) demonstrated that the interactive affinity of virtual streamers positively affects consumers’ purchase intention. Feng et al. (2024) found that, on a subconscious level, the positive emotions displayed by virtual streamers can evoke similar emotions in consumers, thereby enhancing their purchase intention. However, it is important to note that current research on the interactivity of E-commerce live streamers predominantly focuses on either real-life or virtual streamers in isolation, with limited exploration of the differences in interaction between these two types of streamers. Despite existing studies demonstrating the significant impact of streamer interactivity on consumer behavior, there is a lack of direct comparison and analysis of the interaction modes and effects between real-life and virtual streamers (see Table 1 for a summary of the literature in this area).

**Table 1:** Summary of extant research on the interactivity of E-commerce streamers.

Reference	Theoretical perspective	Key findings	Main IV(s)	Med(s)	Main DV(s)	Streamer type	
						Human streamer	Virtual streamer
Chen H. et al. (2024) and Chen T. et al. (2024)	–	Product and social information contained in streamer interactive conversation content has an inverted U-shaped relationship with consumer purchase behavior, while emotional information has a negative linear relationship with consumer purchase behavior.	Streamers’ interactive conversation content		Purchase behavior	√	
Chen and Li (2024)	Expectancy violations theory	Professionalism expectation violation, empathy expectation violation, and responsiveness expectation violation positively influenced consumers’ distrust and dissatisfaction, which subsequently led to discontinuance behavior.	Negative expectation violations	Distrust, Dissatisfaction	Discontinuance behavior		√
Cheng (2022)	SOR theory	Compared with ordinary consumer salutations, cordial consumer salutations have a stronger impact on impulse purchase intentions	Consumer salutation of internet celebrity streamers × Decision-making thinking mode	Perceived social distance,	Impulse purchase intention	√	
Feng (2022)	SOR theory	The interactivity, charisma, and professionalism of live streamers have a positive impact on consumers’ purchase intention.	Interactivity, Charisma, Professionalism	Perceived quality, Perceived risk	Purchase intentions	√	
Feng et al. (2024)	Emotional contagion theory	At subconscious level, the positive emotions exhibited by a virtual streamer can evoke similar emotions in consumers, thereby bolstering their purchase intention; at a conscious level, these positive emotions enhance purchase intention via the mechanism of positive expectation disconfirmation.	Virtual streamer’s positive emotions	Consumer’s positive emotions, positive disconfirmation	Purchase intentions		√
Gao et al. (2023)	Social presence	The popularity, activity level, and responsiveness of virtual streamers enhance both social presence and telepresence, which in turn facilitate purchase intention.	Likeability, animacy, responsiveness	Social presence, telepresence	Purchase intentions		√
Gong and Sun (2025)	Mind perception theory	Emotional language used by virtual streamers is more effective than rational language. Moreover, emotional language leads to higher CIFA, with consumers’ mind perception mediating this link in the livestreaming E-commerce context.	Emotional language vs. Rational language	Perceived agency, perceived experience	Intention to follow the advice		√
He et al. (2024)	–	During online live streaming interactions, the impact of the credibility, professionalism, and attractiveness characteristics of live streamers on consumers’ purchase intention	Trustworthiness, professionalism, attractiveness	Emotional response	Purchase intentions		√
Liao et al. (2023)	Flow theory	Streamers’ interaction orientation has a positive effect on viewers’ immersion and parasocial interactions, in turn positively affecting viewers’ willingness to purchase.	Interaction orientation	Parasocial interaction, audience immersion	Purchase intentions	√	
Luo X. et al. (2024) and Luo L. et al. (2024)	Social support theory	Speech content of streamers can be categorized into informative and affective topics, and these two types of topics have different effects on users’ engagement across behavioral, emotional and relational dimensions.	Informative topics, affective topics	–	Behavioral, emotional and relational engagement	√	
Sun and Tang (2024)	Social response theory	Behavioral realism positively affects consumer purchase intention only when the virtual streamers’ form realism is low.	Form Realism × Behavioral realism	Parasocial Interaction	Purchase intentions		√
Tong et al. (2024)	SOR theory	The affinity of virtual streamers has a positive impact on consumers’ purchase intention. However, the mimicry and responsiveness of virtual streamers do not have a significant impact on consumers’ purchase intention.	Affinity, mimicry, responsiveness	Trust (competence, intimacy)	Purchase intentions		√
Wang K. et al. (2023) and Wang C. et al. (2023)	Social presence	The process of the impact of virtual streamer interactivity on consumers’ purchase intention is fully mediated by social presence. It is also found that product type moderates the aforementioned process.	Interactivity	Social presence	Purchase intentions		√
Wu et al. (2023)	Social response theory	Streamers’ socialness has a positive significant effect on experiential value.	Socialness (high-social vs. low-social)	Social presence	Experiential value		√
Xu et al. (2024)	Social identity theory (SIT), experiential value theory	Personalization, human-like personality, system quality and content quality are positively associated with parasocial interaction and experiential value, which subsequently impact brand image.	Personalization, human-like personality, system quality, content quality	Parasocial interaction, experiential value	Brand image		√
Yao et al. (2024)	SCM	The effect of social-oriented language on purchase intention solely in the experience product condition.	Linguistic style × Product type	Perceived warmth, perceived competence	Purchase intentions		√
Zhang et al. (2024)	Epistemic vigilance	Compared with task-oriented styles, social-oriented interaction styles are more likely to boost consumer engagement, and it also investigates the mediating role of epistemic vigilance and the moderating role of consumer expertise.	Streamer interaction style (task-oriented vs. social-oriented)	Epistemic vigilance	Customer engagement	√	
**This study**	**Social identity theory**	**This paper explores the interactive effects and underlying mechanisms of affectionate nicknames and streamer type on attitudes toward E-commerce streamers, with the mediating mechanism being consumers’ perception of psychological closeness.**	**Affectionate nicknames (use vs. non-use) × Streamer type (human streamer vs. virtual streamer)**	**Psychological closeness**	**Streamer attitude**	√	**√**

The bolded terms are meant to highlight the key aspects of the research in this paper.

### 2.2 Address terms

Despite substantial progress in understanding the dynamics of interactivity between E-commerce streamers and consumers, there is still a limited grasp of the interpersonal and human–machine relationships in these interactions. Address forms, as key elements of first impressions in interpersonal interactions, not only reveal the identity and status of the addressee but also reflect the attitude, thoughts, and sincerity of the addresser (Enfield, 2009; Locher, 2013). These are personal pronoun expressions used by speakers to refer to their conversational counterparts (Brown and Yule, 1983). Research shows that employing kinship terms in non-kinship communication can significantly reduce interpersonal distance (Pan and Liu, 1994); for instance, merchants in southern China commonly use kinship terms to address customers to reduce social distance (Pan, 2000). In northern China, sellers in lower-status markets often use terms like “brother” or “sister,” while high-status retailers prefer deferential terms such as “nin” (polite form of “you”) (Liu, 2009). In E-commerce live streaming, streamers often use “affectionate” language, such as intimate nicknames like “Baobao” (Baby) or “Baozi” (Dear ones), to foster emotional closeness and enhance bonds with viewers (Wang, 2022). Cheng (2022) found that such intimate address forms, compared to standard ones, exert a stronger influence on impulse purchase intentions. While current studies primarily examine the role of address forms in reflecting social norms, cultural backgrounds, and relationship management (Qi, 2011; Clyne et al., 2009), their application in human–machine relationships remains underexplored. This paper addresses this gap by exploring the role of address forms in human–machine relationships and analyzing interaction differences between human and virtual streamers. Specifically, it examines the differential effects of affectionate nicknames on consumer responses through comparative analysis.

## 3 Hypothesis development

### 3.1 Interactive effect of streamer type and affectionate nicknames

Grounded in Social Identity Theory, we hypothesize that consumers’ responses to the use of affectionate nicknames differ based on the type of E-commerce streamer. Specifically, when human streamers use affectionate nicknames, they can convey warmth and kindness (Leech, 2014), thereby enhancing consumers’ positive attitudes toward the streamer. In contrast, avatars, or virtual streamers, as non-human entities, primarily function as assistants and supplementary services to human streamers. The emotional distance in human-avatar interactions, which are primarily transactional in nature, is inherently greater than that in interpersonal relationships. Moreover, when address forms fail to align with the level of intimacy between communicators, they may lead to misunderstandings or negative feelings (Yao, 1991). Therefore, when virtual streamers use affectionate nicknames in live streaming—linguistic cues typically employed by humans to reduce identity distance—it may trigger a sense of identity threat among consumers, thereby eliciting negative emotions. To validate this hypothesis, this study will investigate the efficacy of affectionate nicknames across different streamer types and explore the underlying psychological mechanisms through experimental design, data collection, and analysis.

### 3.2 Mediating role of psychological closeness

Psychological closeness as defined by Gino and Galinsky (2012), refers to the perceived sense of attachment and connection to another entity. This concept derives from the broader notion of psychological distance, which encompasses “a range of subjective experiences related to proximity to or distance from a person, place, event, or psychological construct” (Williams et al., 2014). When consumers experience psychological closeness with others, they tend to be less suspicious of ulterior motives in communication and behavior, and more receptive to friendly requests (Ren et al., 2018; Williams et al., 2014). Aron and Aron’s (1986) research suggests that individuals who feel psychologically close to another person are less likely to question the latter’s intentions, often perceiving their actions as well-intentioned and warm. As a result, psychological closeness can elicit positive emotional responses and behaviors in consumers, influencing their perceptions, attitudes, and decision-making processes (Williams et al., 2014). In essence, the presence or absence of psychological closeness between consumers and E-commerce streamers is critical in determining consumer attitudes toward the streamer.

**Figure 1:**
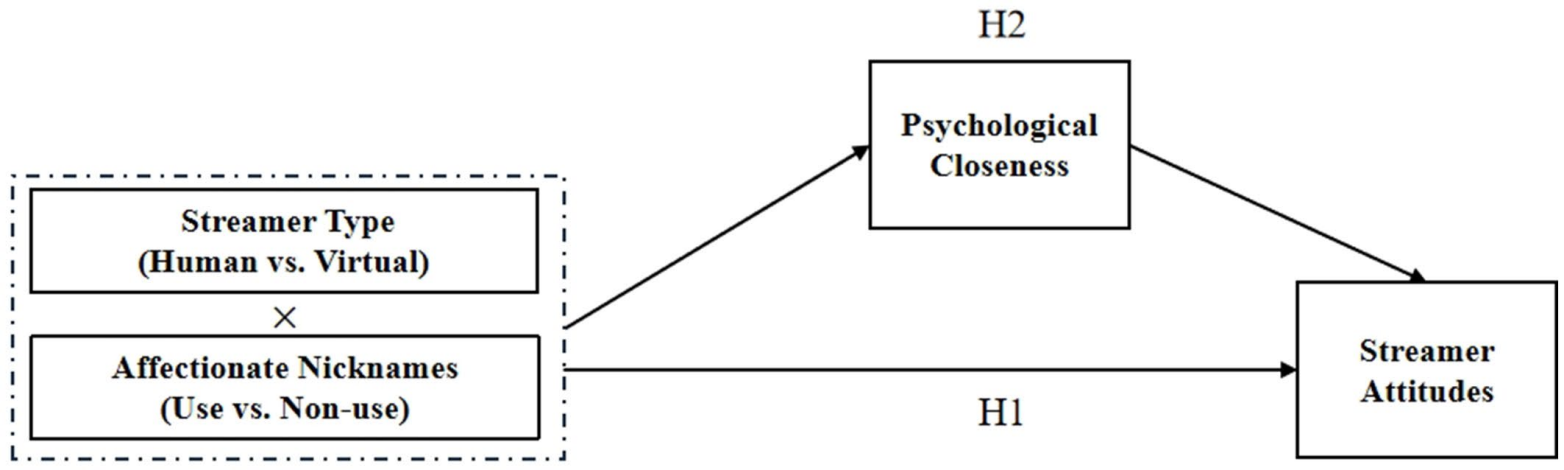
Hypothetical model.

Within the domain of E-commerce live streaming, the streamer-consumer relationship is primarily rooted in social identity. The streamer type significantly influences the level of psychological closeness consumers experience. Specifically, in scenarios involving human streamers, consumers and streamers share a common in-group identity (as humans), leading to more favorable evaluations — a phenomenon known as in-group favoritism. As such, the use of affectionate nicknames by human streamers in E-commerce live streaming promotes psychological closeness between the streamer and consumers, compared to situations where such nicknames are absent. This, in turn, enhances consumer attitudes toward the streamer. By contrast, in the context of virtual streamers, consumers often perceive these digital assistants as members of an out-group (non-humans). The use of in-group affectionate nicknames in this setting may trigger intergroup biases (Pettigrew, 1979; Taylor and Jaggi, 1974), resulting in hostility or derogation toward the out-group. Therefore, in the case of virtual streamers in E-commerce live streaming, refraining from using affectionate nicknames may instead foster psychological closeness between the streamer and consumers, thereby improving consumer attitudes toward the streamers. The hypothetical model is shown in Figure 1.

Drawing from this analysis, the following hypotheses are proposed:

*H2*: Psychological closeness plays a mediating role in the effect described in H1.

*H2a*: The use (vs. non-use) of affectionate nicknames is more likely to enhance consumer attitudes toward the human streamer by fostering psychological closeness.

*H2b*: The non-use (vs. use) of affectionate nicknames is more likely to enhance consumer attitudes toward the virtual streamer by fostering psychological closeness.

## 4 Methodology

### 4.1 Measures

Responses to the items listed below, adapted from established measures, were utilized to construct the study’s measures and evaluate the hypothesized structural model. Response options for each item were measured on a 7-point Likert scale, ranging from 1 (strongly disagree) to 7 (strongly agree). Streamer type was measured using items adapted from Zhou et al. (2024), Wang K. et al. (2023), and Wang C. et al. (2023). Affectionate nicknames were evaluated through items adapted from Wang (2022). Streamer attitude was measured using items adapted from Bhattacherjee and Sanford (2006). Psychological closeness was measured through items adapted from Gino and Galinsky (2012). The items are summarized in Table 2.

**Table 2:** Questionnaire items.

Constructs	Items	Literature
Streamer type	To what extent do you believe that the streamer is a real person (or a virtual streamer)?	Zhou et al. (2024), Wang K. et al. (2023), and Wang C. et al. (2023)
Affectionate nicknames	I feel that this term carries an emotional connotation of affection and closeness, and conveys an effort to build closer interpersonal relationships.	Wang (2022)
Streamer attitude	I think watching this streamer’s live stream is a good choice.	Bhattacherjee and Sanford (2006)
	I think watching this streamer is enjoyable.	
	I think watching this streamer’s live stream feels good.	
Psychological closeness	I feel very close to this streamer.	Gino and Galinsky (2012)
	I feel a close relationship with this streamer.	
	My unfamiliarity with this streamer has decreased.	

### 4.2 Sampling and data collection procedures

Online surveys for all five experiments in this study were conducted using *Credamo*,^1^ a comprehensive research platform offering services including questionnaire design, participant sampling, and statistical modeling. *Credamo* features a specialized tool for designing randomized scenario-based experiments. Specifically, the platform incorporates a random questionnaire module, allowing scenario-based modules to be customized to meet experimental requirements. This feature ensures that participants encounter varied experimental scenarios upon accessing the questionnaire, enabling random assignment to distinct experimental groups. Beyond scenario-specific modules, the remaining questionnaire components, including demographic information, are standardized across all participants.

In the pretest experiment, 100 participants (61% female) were recruited to evaluate their perception of affectionate nicknames after reviewing explanatory materials. Prior to the experiments, participants’ familiarity with E-commerce live streaming as a shopping format was confirmed. In Experiment 1, a pretest with 38 participants (74% female) randomly assigned to four groups tested manipulations of streamer type and affectionate nicknames. The main experiment involved 368 participants (61% female), who were randomly allocated to four groups to test H1 using images and textual descriptions of E-commerce live streaming rooms. Similarly, Experiment 2 included a pretest with 40 participants (60% female) and a main experiment with 370 participants (61% female) to test H2 using videos of E-commerce live streaming rooms. A total of 96 valid questionnaires were collected. The participants were 62% female, with 89% under the age of 40, consistent with industry data on E-commerce live streaming in China.

As the independent and moderating variables in our model are categorical and the dependent variable is continuous, two-way ANOVA was employed for analysis. To further investigate the impact of affectionate nicknames on consumers’ streamer attitude and psychological closeness across different streamer types, simple effect analyses were performed. To examine the mediating effect of psychological closeness, moderated mediation analysis was conducted using Hayes (2013) PROCESS Model 8 with 5,000 bootstrapped samples and a 95% confidence interval. Finally, participants’ gender, age, occupation, education level, and income were included as covariates in the ANOVA to control for potential confounding effects.

## 5 Materials and methods

### 5.1 Pretest

This study primarily employs a situational experimental method for data collection. Compared to cross-sectional surveys or secondary data analysis, experimental methods allow for the manipulation of independent variables before measuring dependent ones. This approach can partially mitigate the endogenous issue of mutual causality, thus facilitating a more accurate examination of causal relationships (Chen et al., 2012; Zhang and Wang, 2022). The specific methodologies and results of each experiment are detailed below.

Prior to the formal experiment, an independent pretest was conducted to determine the effectiveness of the experimental material design, particularly in relation to affectionate nicknames. Through a comprehensive review of relevant literature on address forms in E-commerce live streaming environments, and the collation of address forms from popular live streaming platforms such as Taobao, TikTok, and Kuaishou, a total of 18 common address forms were identified.

Based on the semantic interpretation of affectionate nicknames and the emotional expression characteristics demonstrated by streamers in E-commerce live streaming (Wang, 2022), 100 participants (61% female; 95% aged under 40 years) were recruited via *Credamo*. These participants were tasked with rating their perception of affectionate nicknames on a scale of 1–7, with 1 representing “strongly disagree” and 7 representing “strongly agree.” This rating was conducted after reviewing materials that defined affectionate nicknames.

The review material presented to participants was as follows: “In accordance with the definition provided in the ‘Modern Chinese Dictionary’, affectionate nicknames convey meanings similar to ‘affectionate address’ and ‘pet name.’ In everyday interactions, such nicknames are commonly used to express sentiments of closeness and fondness. Similarly, within the context of live streaming rooms on platforms such as Taobao, JD.com, TikTok, and Kuaishou, streamers use these nicknames to greet their viewers. Therefore, we request that you evaluate whether the following address forms used in E-commerce live streaming rooms ‘convey a sense of warmth and closeness, while also attempting to bridge interpersonal gaps.’ Please indicate your level of agreement on a scale from 1 (‘strongly disagree’) to 7 (‘strongly agree’).”

The assessment of affectionate nickname terms revealed that “Jiarenmen” (Family), “Baozimen” (Dear ones), and “Baobaomen” (Babies) garnered the highest scores in terms of affectionate nickname perception. Conversely, “Dajia” (Everyone), “Nvshimen” (Ladies), and “Xianshengmen” (Gentlemen) exhibited the lowest perception of affection. The outcomes are clearly presented in the adjoining table. Based on these findings, this study opted to utilize the highly rated “Baobaomen” (Babies), “Baozimen” (Dear ones), and “Baobeimen” (Precious ones) as the affectionate nicknames for the forthcoming experiments (see Table 3).

**Table 3:** Analysis of data on perception of affectionate nicknames used by E-commerce streamers.

Affectionate nicknames	Sample size	Minimum	Maximum	Average	Standard deviation	Median
Family (Jiarenmen)	100	2	7	5.98	0.943	6
Dear Ones (Baozimen)	100	1	7	5.8	1.206	6
Babies (Baobaomen)	100	2	7	5.75	1.167	6
Buddies (Tietiemen)	100	2	7	5.73	1.062	6
Precious Ones (Baobeimen)	100	1	7	5.47	1.283	6
Dear Ones (Qinrenmen)	100	1	7	5.35	1.359	6
Sister (Xiaojiejie)	100	1	7	5.27	1.355	5
Friends (Pengyoumen)	100	1	7	5.2	1.214	5
Brother (Xiaogege)	100	1	7	5.17	1.256	5
Buddy (Xiaohuoban)	100	2	7	5.16	1.261	5
Sweetheart (Xiaobaobei)	100	1	7	5.02	1.517	5.5
Gorgeous Guy (Shuaige)	100	1	7	4.66	1.539	5
Pretty Girl (Meinv)	100	1	7	4.63	1.468	5
Fans (Fensimen)	100	1	7	4.18	1.592	4
Cute Girl (Meimei)	100	1	7	4.09	1.491	4
Everyone (Dajia)	100	1	7	4.01	1.667	4
Ladies (Nvshimen)	100	1	7	3.87	1.721	4
Gentlemen (Xianshengmen)	100	1	7	3.79	1.748	4

The average represents the central tendency of participants’ perception of the nickname. A higher average indicates a greater perceived intimacy toward the nickname among participants. The standard deviation indicates the extent to which participants’ ratings deviate from the mean, reflecting variability in their perception of the nickname. A larger standard deviation signifies higher variability in participants’ ratings of the nickname.

### 5.2 Study 1

#### 5.2.1 Pretest

The pretest employed a 2 (Affectionate Nicknames: use vs. non-use) × 2 (Streamer Type: Human Streamer vs. Virtual Streamer) between-subjects design. The manipulation of real and virtual streamers was inspired by Zhou et al. (2024), and the streamer type was validated through participants’ ratings of the perceived streamer image on a scale from 1 (representing a virtual streamer) to 7 (representing a human streamer). The use of affectionate nicknames was manipulated through the inclusion or omission of the term “Babies” in the greeting. Subsequently, participants were asked to rate the degree of affectionate perception on a scale ranging from 1 (no affectionate nickname) to 7 (affectionate nickname present). In the affectionate nickname condition, the streamer greeted new viewers to the live stream with “Babies, hello, welcome to our live stream.” Conversely, in the absence of affectionate nicknames, the streamer simply said, “Hello, welcome to our live stream.” Additionally, the pretest evaluated the authenticity and comprehensibility of the materials. Participants were required to indicate the realism of the scenario and their comprehension of the information, on a scale from 1 (completely unrealistic/incomprehensible) to 7 (completely realistic/comprehensible).

A total of 38 participants (70% female; 84% aged under 40 years) were recruited through *Credamo* and randomly assigned to one of four groups. A two-way ANOVA revealed that the perceived authenticity of human streamers was significantly higher than that of virtual streamers [*M*_human_ = 5.94, *M*_virtual_ = 1.57, *F*(1,36) = 239.266, *p* < 0.001]. Similarly, the mean rating for affectionate nicknames was significantly higher when affectionate nicknames were used compared to when they were not [*M*_use_ = 6.09, *M*_non-use_ = 2.25, *F*(1,36) = 21.339, *p* < 0.001]. The main effect of streamer type on streamer perception was significant [*F*(1,36) = 239.266, *p* < 0.001], whereas the main effect of affectionate nicknames on streamer perception was non-significant [*F*(1,36) = 0.045, *p* = 0.834]. The interaction between streamer type and affectionate nicknames was similarly non-significant [*F*(1,36) = 0.980, *p* = 0.329].

Regarding the manipulation of affectionate nicknames, the mean rating was significantly higher when affectionate nicknames were used (*M*_use_ = 6.09, *M*_non-use_ = 2.25), and the main effect of streamer type on perceptions of affectionate nicknames was non-significant [*F*(1,36) = 0.037, *p* = 0.850]. Conversely, the main effect of affectionate nicknames on perceptions of affectionate nicknames was highly significant [*F*(1,36) = 201.339, *p* < 0.001], while the interaction between streamer type and affectionate nicknames remained non-significant [*F*(1,36) = 0.182, *p* = 0.672]. Moreover, the mean ratings for material authenticity (*M*_authenticity_ = 5.842) and comprehensibility (*M*_comprehensibility_ = 6.026) were each significantly higher than 4, indicating the materials were both realistic and understandable. Therefore, the formal experiment is justified based on these pretest findings.

#### 5.2.2 Participants

The purpose of Study 1 was to examine the interactive effects of streamer type and affectionate nicknames on streamer attitude and specifically to test whether Hypothesis 1 is supported. The manipulations and procedures of the formal experiment were largely consistent with those of the pilot study. Through *Credamo*, a total of 368 valid samples were collected for the experiment. The study included 93 participants in the group where human streamers used affectionate nicknames, 91 in the group where human streamers did not use affectionate nicknames, 92 in the group where virtual streamers used affectionate nicknames, and 92 in the group where virtual streamers did not use affectionate nicknames.

According to data from industry research reports on Chinese E-commerce live streaming platforms, female consumers represent the majority group on platforms such as Taobao Live and TikTok Live, with over 60% of the audience, a proportion significantly higher than that of male consumers. The gender distribution of participants recruited for both the pilot and formal experiments aligns with this industry-wide trend. Descriptive statistics of the sample are presented in Table 4.

**Table 4:** Sample descriptive statistics (*N* = 368).

Variable	Category	Frequency	Proportion (%)
Gender	Female	225	61.14
	Male	143	38.86
Age group	0–20 years old	28	7.61
	21–30 years old	160	43.48
	31–40 years old	127	34.51
	41–50 years old	29	7.88
	51–60 years old	22	5.98
	Over 60 years old	2	0.54
Occupation	Public Institution	20	5.43
	Government Employee	16	4.35
	State-owned Enterprise	47	12.77
	Foreign-funded Enterprise	12	3.26
	Student	77	20.92
	Private Enterprise	196	53.26
Education level	Junior High School	1	0.27
	High School/Vocational School	9	2.45
	College	30	8.15
	Bachelor’s Degree	268	72.83
	Master’s Degree	52	14.13
	Doctor’s Degree	8	2.17
Monthly income	3,000–5,000	54	14.67
	Below 3,000	67	18.21
	5,001–8,000	96	26.09
	Above 8,000	151	41.03
Total		368	100

The table summarizes the demographic characteristics of the sample population (*N* = 368), including variables such as gender, age group, occupation, education level, and monthly income level. Frequencies and proportions (%) are provided for each category within each variable.

#### 5.2.3 Procedure

Before the experiment, participants were initially asked if they had watched live streams on E-commerce live streaming platforms. They were subsequently assigned randomly through *Credamo* to one of the four experimental groups. Participants were instructed to imagine that they were preparing to purchase a pair of sunglasses and had entered the live streaming room of a virtual sunglasses brand called “MOPENG” and were greeted by the streamer. Based on pretest materials, in the live streaming rooms where affectionate nicknames were used, the streamer addressed participants as “Babies.” In rooms where affectionate nicknames were not used, the streamer did not use affectionate address forms.

In the human streamer group, participants saw images of real people, whereas in the virtual streamer group, they saw images of avatars. Subsequently, participants were required to answer manipulation check questions regarding affectionate nicknames and streamer types. Finally, participants responded to questions about streamer attitude (Cronbach’s *α* = 0.907) (Bhattacherjee and Sanford, 2006), attention checks, and demographic questions, and were subsequently compensated. The streamer attitude measurement consisted of three items: “I think watching this streamer’s live stream is a good choice,” “I think watching this streamer is enjoyable,” and “I think watching this streamer’s live stream feels good.” The scale used was a 7-point Likert scale, ranging from 1 (“strongly disagree”) to 7 (“strongly agree”) (Bhattacherjee and Sanford, 2006).

#### 5.2.4 Data results

##### 5.2.4.1 Manipulation checks

The results of a two-way ANOVA showed that the mean perceived authenticity for real streamers was significantly higher than that for virtual streamers (*M*_human_ = 6.28, *M*_virtual_ = 1.69). The main effect of streamer type on perceived streamer type was significant [*F*(1,366) = 3295.875, *p* < 0.001], while the main effect of affectionate nicknames on perceived streamer type was not significant [*F*(1,366) = 0.0413, *p* = 0.521]. The interaction effect between streamer type and affectionate nicknames was not significant [*F*(1,366) = 0.339, *p* = 0.561]. The mean evaluation of affectionate nicknames for the group using affectionate nicknames was higher than that for the group not using affectionate nicknames (*M*_use_ = 6.20, *M*_non-use_ = 1.72). The main effect of streamer type on the perception of affectionate nicknames was not significant [*F*(1,366) = 0.913, *p* = 0.340], while the main effect of affectionate nicknames on the perception of affectionate nicknames was significant [*F*(1,366) = 2983.507, *p* < 0.001]. The interaction effect between streamer type and affectionate nicknames was not significant [*F*(1,366) = 0.987, *p* = 0.321]. Both streamer type and affectionate nickname manipulations were successfully validated.

##### 5.2.4.2 Interaction effect analysis

**Figure 2:**
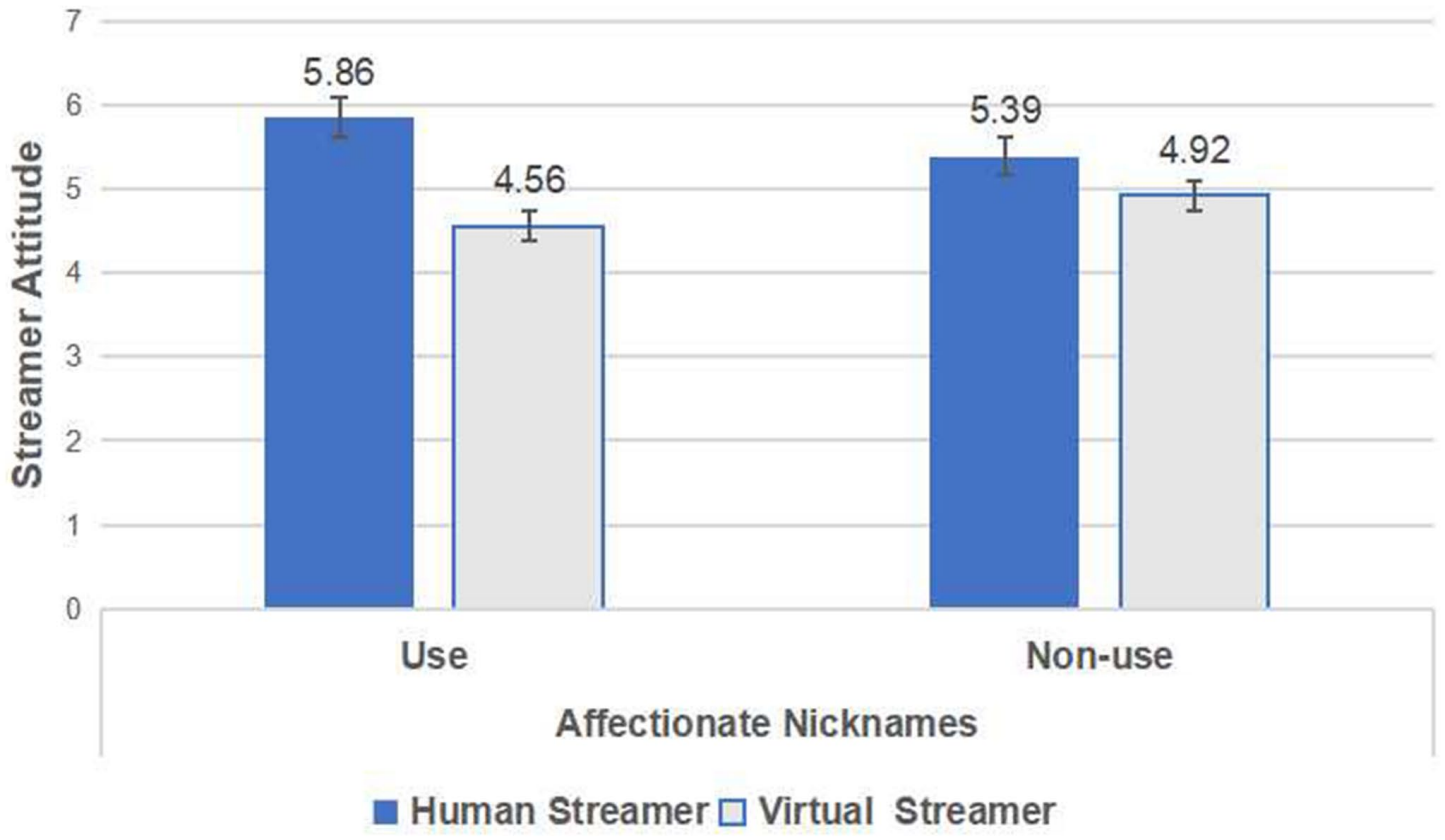
The interactive effect of streamer type and affectionate nicknames on streamer attitude in Study 1.

Using streamer attitude as the dependent variable, and streamer type and affectionate nicknames as independent variables, it was found that the interaction between streamer type and affectionate nicknames exerted a significant effect on streamer attitude [*F*(1,366) = 13.485, *p* < 0.001]. The effect of affectionate nicknames on consumers’ perceptions of streamer attitude was not significant [*F*(1,366) = 0.233, *p* = 0.630], while the influence of streamer type on streamer attitude was significant [*F*(1,366) = 6.731, *p* < 0.001]. To further explore the impact of affectionate nicknames on consumers’ streamer attitudes within different streamer types, a simple effects analysis was conducted. The results showed that in the real streamer group, using affectionate nicknames (*M*_use_ = 5.86, SD = 0.72) led to a more positive streamer attitude than not using affectionate nicknames (*M*_non-use_ = 5.39, SD = 1.09) [*F*(1,182) = 12.123, *p* = 0.001 < 0.01], confirming H1a. In the virtual streamer group, using affectionate nicknames (*M*_use_ = 4.56, SD = 1.18) led to a less positive streamer attitude than not using affectionate nicknames (*M*_non-use_ = 4.92, SD = 1.29) [*F*(1,182) = 3.950, *p* = 0.048 < 0.05], confirming H1b, as shown in Figure 2. Additionally, accounting for the potential effects of participants’ gender, age, occupation, education level, and income on the results, these variables were treated as covariates in subsequent ANOVA analysis. The results indicated that the interaction between streamer type and affectionate nicknames continued to exert a significant effect on consumers’ streamer attitude [*F*(1,366) = 11.605, *p* < 0.01, *η*^2^ = 0.031].

##### 5.2.4.3 Discussion

Study 1 examined the interactive effect of streamer type and affectionate nicknames on consumers’ attitudes toward streamers. Specifically, when consumers interact with human streamers, the use of affectionate nicknames is more likely to elicit positive attitudes toward the streamer compared to their absence. Conversely, when consumers encounter virtual streamers, the absence of affectionate nicknames tends to provoke more negative attitudes compared to their presence. Although Study 1 confirmed the effects of Hypothesis H1, there are still several limitations that must be addressed. Firstly, the experimental materials in Study 1 were image-based; however, in actual E-commerce live streaming consumers engage with dynamic streamer greetings and explanations. Secondly, Study 1 did not investigate the underlying mechanisms of the interaction between streamer type and affectionate nicknames on consumers’ attitudes toward streamers. To address these limitations, we designed Study 2 to further explore these issues.

### 5.3 Study 2

#### 5.3.1 Pretest

The pretest employed a 2 × 2 between-subjects design with factors of affectionate nicknames (Use vs. Non-use) and streamer type (Human Streamer vs. Virtual Streamer). The experimental manipulation was based on the study by Wang K. et al. (2023) and Wang C. et al. (2023). The manipulation of virtual streamers was signaled by the label “virtual streamer” in the live streaming room, whereas no such label was used for human streamers. The manipulation of affectionate nicknames involved greetings such as “babies” or “dear ones” being used in the live streaming room. In the video, the streamer greeted the participants, introduced products, and recommended the live streaming room. Participants were randomly assigned via *Credamo* to one of the four experimental conditions and were instructed to imagine browsing products in the live streaming room before completing the corresponding questionnaire based on their genuine impressions. The pilot experiment assessed streamer type and affectionate nicknames using the same methods as Study 1. Additionally, it evaluated the authenticity and comprehensibility of the materials.

A total of 40 participants (60% female; 90% aged under 40 years) were randomly recruited via *Credamo* for the pilot experiment and were subsequently assigned to one of four groups. The results of a two-way ANOVA revealed that the mean perception of human streamers was significantly higher compared to that of virtual streamers (*M*_human_ = 5.75, *M*_virtual_ = 1.75). The main effect of streamer type on streamer type perception was significant [*F*(1,38) = 83.965, *p* < 0.001], whereas the main effect of affectionate nicknames on streamer type perception was not significant [*F*(1,38) = 0.000, *p* = 1.000]. The interaction between streamer type and affectionate nicknames was also non-significant [*F*(1,38) = 2.571, *p* = 0.118]. The mean evaluation of affectionate nicknames in the group that used them was higher compared to the group that did not use them (*M*_use_ = 6.35, *M*_non-use_ = 1.45). The main effect of streamer type on the perception of affectionate nickname usage was non-significant [*F*(1,38) = 0.157, *p* = 0.695], whereas the main effect of affectionate nicknames was significant [*F*(1,38) = 375.809, *p* < 0.001]. The interaction between streamer type and affectionate nicknames was non-significant [*F*(1,38) = 0.626, *p* = 0.434]. Meanwhile, the mean values for material authenticity (*M*_authenticity_ = 5.975) and comprehensibility (*M*_comprehensibility_ = 6.050) were both significantly greater than 4. Based on these findings, a formal experiment was subsequently conducted.

#### 5.3.2 Participants

The purpose of Study 2 was to investigate the mediating role of psychological closeness, further examining the mechanism by which streamer type and affectionate nicknames influence consumers’ perceptions of streamer attitudes and to provide a robustness check of the findings from Study 1. Consistent with the pilot experiment, a 2 (Affectionate Nicknames: Use vs. Non-use) × 2 (Streamer Type: Human Streamer vs. Virtual Streamer) between-subjects experimental design was utilized. A total of 370 valid questionnaires were collected via *Credamo*, with 94 participants in the human streamer with affectionate nicknames group, 92 in the human streamer without affectionate nicknames group, 93 in the virtual streamer with affectionate nicknames group, and 91 in the virtual streamer without affectionate nicknames group. The descriptive statistics of the sample are shown in Table 5.

**Table 5:** Sample descriptive statistics (*N* = 370).

Variable	Category	Frequency	Proportion (%)
Gender	Female	227	61.35
Age group	Male	143	38.65
	0–20 years old	19	5.14
	21–30 years old	208	56.22
	31–40 years old	108	29.19
	41–50 years old	23	6.22
	51–60 years old	12	3.24
Occupation type	Public Institution	22	5.95
	Civil Servant	11	2.97
	State-owned Enterprise	44	11.89
	Foreign-funded Enterprise	21	5.68
	Student	88	23.78
	Private Enterprise	184	49.73
Education level	Primary School & Below	1	0.27
	High School/Vocational School/Technical School	9	2.43
	Junior College	31	8.38
	Bachelor’s Degree	264	71.35
	Master’s Degree	59	15.95
	Doctoral Degree	6	1.62
Monthly income	3,000–5,000	74	20
	Below 3,000	69	18.65
	5,001–8,000	85	22.97
	Above 8,000	142	38.38
Total		370	100

This table summarizes the demographic characteristics of the sample population (*N* = 370), including gender, age group, occupation type, education.

#### 5.3.3 Procedure

Before the experiment, participants were screened to confirm that they had experience watching live streams on E-commerce live streaming platforms. Subsequently, they were randomly assigned via *Credamo* to one of four experimental groups. Participants were asked to imagine themselves casually browsing through an E-commerce live streaming room on a particular platform and encountering a shampoo product live stream. In the live streams where affectionate nicknames were used, the streamer addressed the participants as “babies,” “precious,” and “baby” while introducing products and recommending brands. In contrast, in live streams without the use of affectionate nicknames, the streamer promoted products and brands without employing any address forms. In the human streamer live streams, no streamer type was specified, while in the virtual streamer live streams, the streamer was labeled as a “virtual streamer.” Participants were then required to complete manipulation check questions regarding affectionate nicknames and streamer type. Finally, participants responded to questions regarding streamer attitude (Cronbach’s *α* = 0.882) (Bhattacherjee and Sanford, 2006), psychological closeness (Cronbach’s *α* = 0.874) (Gino and Galinsky, 2012), attention detection, demographics, and other related items. Compensation was provided upon completion. The measurement of streamer attitude was consistent with that in Study 1, whereas the measurement of psychological closeness included three items: “I feel very close to this streamer,” “I feel a close relationship with this streamer,” and “My unfamiliarity with this streamer has decreased” (utilizing a Likert 7 – point scale; 1 = strongly disagree, 7 = strongly agree) (Gino and Galinsky, 2012).

#### 5.3.4 Data results

##### 5.3.4.1 Manipulation check

The results of the two-way ANOVA indicated that the mean perception of realness for human streamers was significantly higher than that for virtual streamers (*M*_human_ = 6.55, *M*_virtual_ = 1.68). The main effect of streamer type on streamer perception was significant [*F*(1,368) = 3093.263, *p* < 0.001], while the main effect of affectionate nicknames on streamer perception was not significant [*F*(1,368) = 0.023, *p* = 0.880]. The interaction between streamer type and affectionate nicknames was also not significant [*F*(1,368) = 0.011, *p* = 0.915]. The mean evaluation of affection for the group that used affectionate nicknames was higher than that for the group that did not (*M*_use_ = 6.01, *M*_non-use_ = 2.30). The main effect of streamer type on affectionate nickname perception was not significant [*F*(1,368) = 0.094, *p* = 0.759], while the main effect of affectionate nicknames on affectionate nickname perception was significant [*F*(1,368) = 795.402, *p* < 0.001]. The interaction between streamer type and affectionate nicknames was again found to be not significant [*F*(1,368) = 0.399, *p* = 0.528]. Both streamer type and affectionate nicknames were successfully manipulated.

##### 5.3.4.2 Streamer attitude

**Figure 3:**
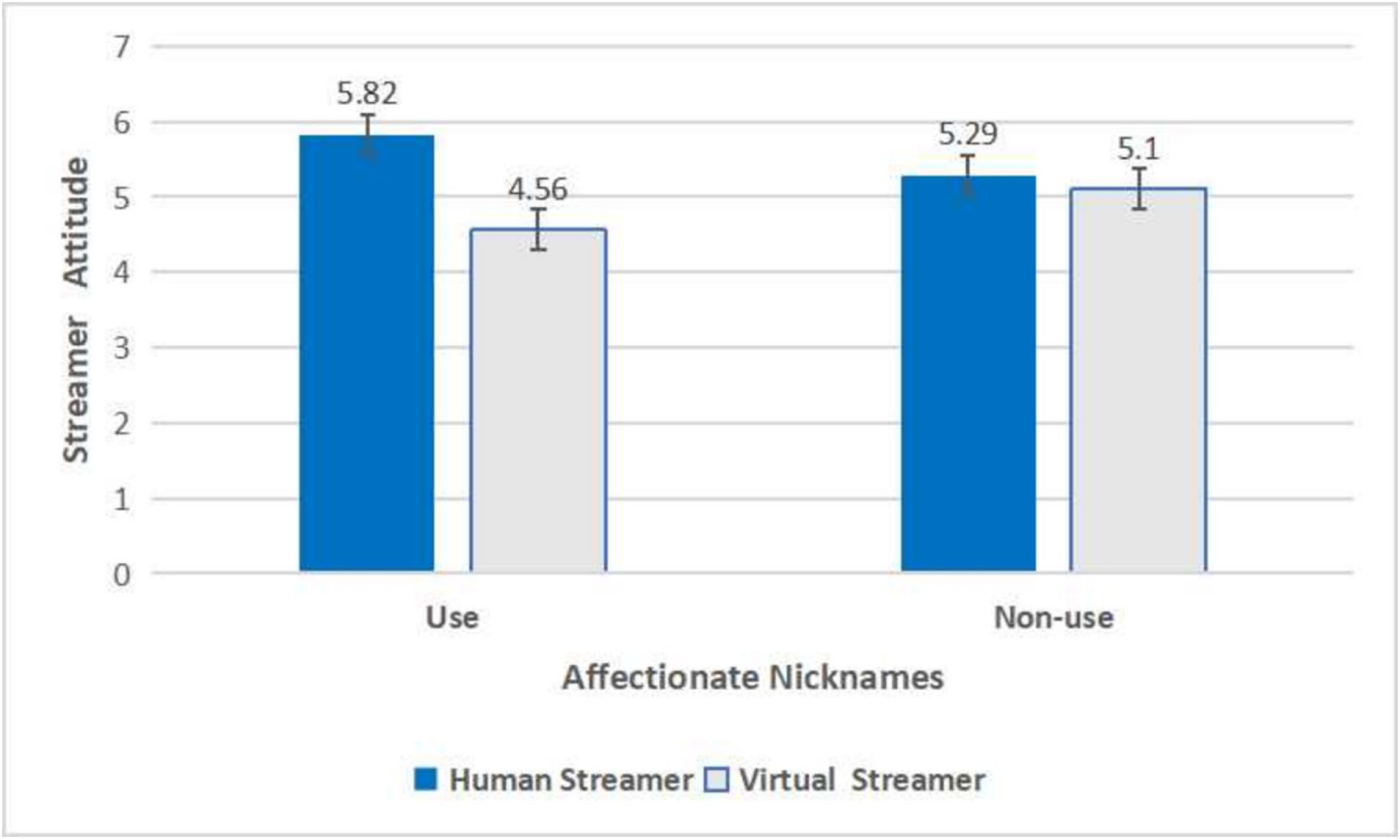
The interactive effect of streamer type and affectionate nicknames on streamer attitude in Study 2.

A two-way ANOVA was conducted with streamer attitude as the dependent variable and streamer type and affectionate nicknames as independent variables, revealing a significant interaction effect between them [*F*(1,368) = 24.545, *p* < 0.001]. In the human streamer group, the use of affectionate nicknames (*M*_use_ = 5.82, SD = 0.78) resulted in a higher streamer attitude compared to not using affectionate nicknames (*M*_non-use_ = 5.29, SD = 0.85) [*F*(1,184) = 19.324, *p* < 0.001], supporting H1a. In the virtual streamer group, the use of affectionate nicknames (*M*_use_ = 4.56, SD = 1.27) resulted in a lower streamer attitude compared to not using affectionate nicknames (*M*_non-use_ = 5.10, SD = 1.16) [*F*(1,182) = 9.065, *p* = 0.003], supporting H1b. Additionally, to control for the potential confounding effects of participants’ gender, age, occupation, education level, and income, these variables were included as covariates in a subsequent ANOVA. The results showed that the interaction effect of streamer type and affectionate nicknames on consumers’ streamer attitude remained significant [*F*(1,368) = 22.361, *p* < 0.001, *η*^2^ = 0.058] (see Figure 3).

##### 5.3.4.3 Psychological closeness

**Figure 4:**
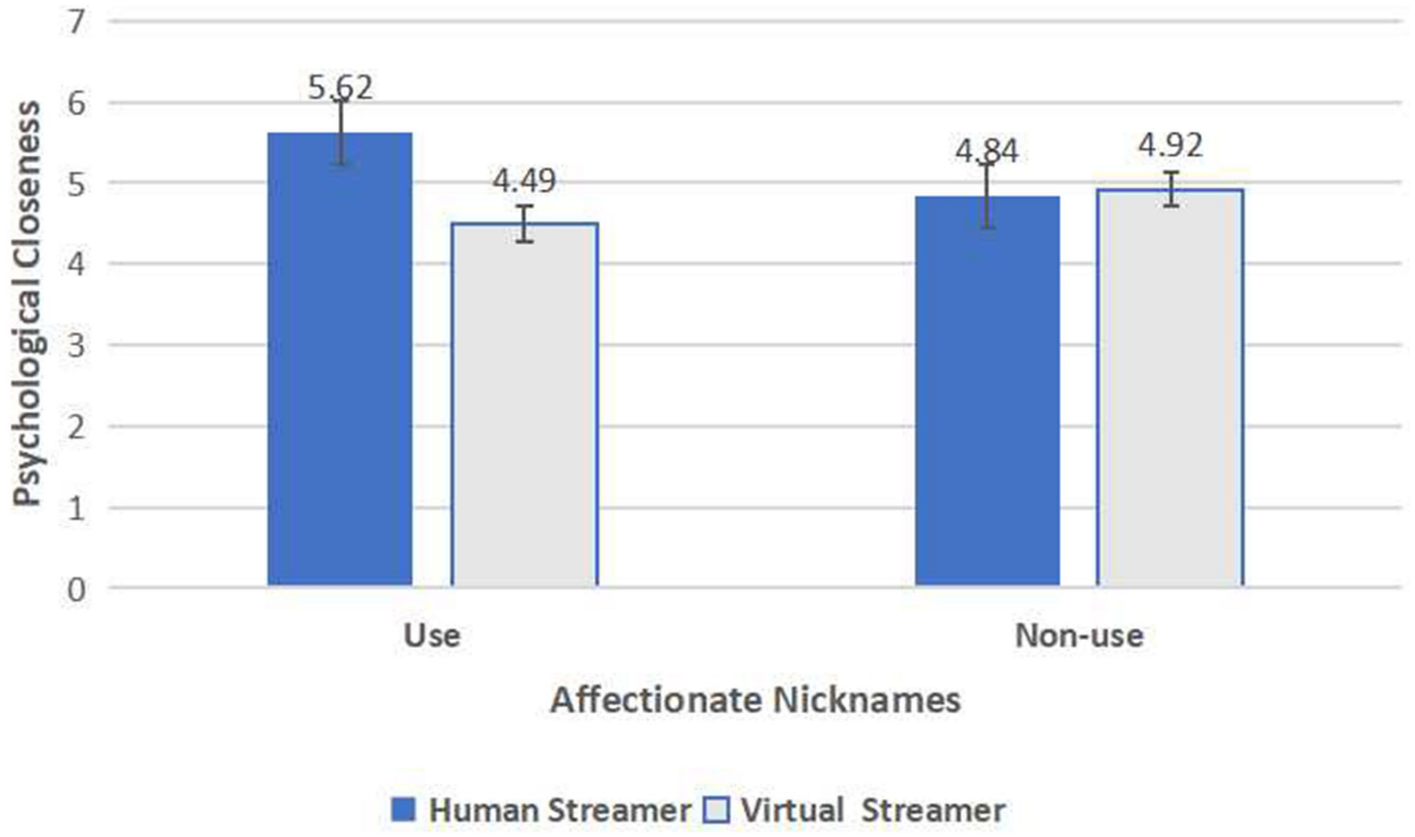
The interactive effect of streamer type and affectionate nicknames on psychological closeness.

A two-way ANOVA was conducted with psychological closeness as the dependent variable and streamer type and affectionate nicknames as independent variables, revealing a significant interaction effect [*F*(1,368) = 23.774, *p* < 0.001]. In the human streamer group, the use of affectionate nicknames (*M*_use_ = 5.62, SD = 0.82) led to higher perceived psychological closeness compared to not using affectionate nicknames (*M*_non-use_ = 4.84, SD = 1.07) [*F*(1,184) = 31.183, *p* < 0.001]. In the virtual streamer group, the use of affectionate nicknames (*M*_use_ = 4.49, SD = 1.49) resulted in lower perceived psychological closeness compared to not using affectionate nicknames (*M*_non-use_ = 4.92, SD = 1.27) [*F*(1,182) = 4.319, *p* = 0.039 < 0.05]. Additionally, to control for the potential confounding effects of participants’ gender, age, occupation, education level, and income, these variables were included as covariates in a subsequent ANOVA. The results showed that the interaction effect of streamer type and affectionate nicknames on psychological closeness [*F*(1,368) = 21.729, *p* < 0.001, *η*^2^ = 0.057] (see Figure 4).

##### 5.3.4.4 Mediating effect analysis

**Figure 5:**
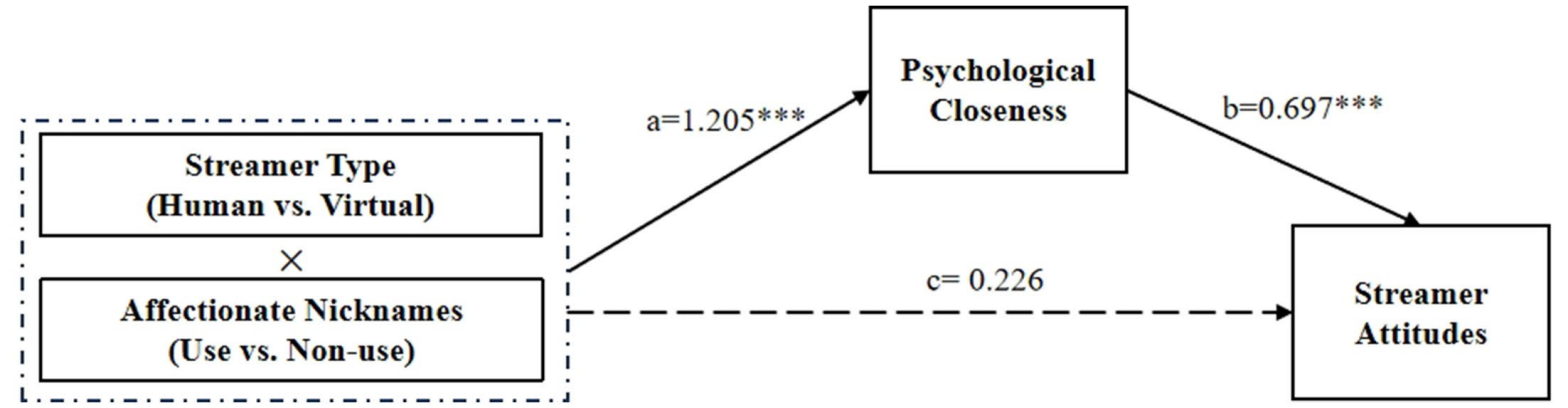
The mediating role of psychological closeness. *p < 0.05, **p < 0.01, ***p < 0.001.

Hayes PROCESS Model 8 with 5,000 bootstrapped samples with a 95% confidence interval (Hayes, 2013) was used to test psychological closeness as a mediator between affectionate nicknames (IV) and streamer attitude (DV). Here, ‘a’ represents the effect of the interaction between streamer type and affectionate nicknames on psychological closeness while ‘b’ reflects the effect of psychological closeness on streamer attitude. The findings suggest that psychological closeness significantly mediates the relationship when acting as a mediator variable (*b* = 0.84, SE = 0.173, 95% CI = [0.500, 1.179]). Specifically, for human streamers, the indirect effect of the interaction between streamer type and affectionate nicknames on streamer attitude through psychological closeness was significant (*b* = −0.544, SE = 0.100, 95% CI = [−0.746, −0.350]). For virtual streamers, the indirect effect of the same interaction on streamer attitude through psychological closeness was also significant (*b* = 0.296, SE = 0.104, 95% CI = [0.223, 0.575]). The interaction effect between streamer type and affectionate nicknames on psychological closeness was 1.205, with a 95% CI of [0.719, 1.691]. The effect of psychological closeness on streamer attitude was 0.697, with a 95% CI of [0.643, 0.751]. Hypothesis 2 was confirmed. Finally, the direct effect of the interaction between streamer type and affectionate nicknames on streamer attitude was not significant, with an effect size ‘c’ of 0.226 and a 95% CI of [−0.036, 0.488] (see Figure 5).

##### 5.3.4.5 Discussion

Firstly, we conducted a robustness test for the effects observed in Study 1 by varying the manipulation of product type and streamer type. Secondly, we found that the interaction between streamer type and affectionate nicknames on consumers’ streamer attitude is mediated by psychological closeness, thereby confirming Hypothesis 2. Specifically, in the human streamer context, the use (vs. non-use) of affectionate nicknames promotes greater psychological closeness, which in turn fosters a more positive attitude toward the streamer. In the virtual streamer context, the non-use (vs. use) of affectionate nicknames leads to greater psychological closeness, thereby fostering a more favorable streamer attitude.

## 6 Results

Drawing on social identity theory, we uncovered the interaction between streamer type and affectionate nicknames, as well as their underlying mechanisms, through two experiments. Specifically, our findings suggest that: (1) There is an interaction between streamer type and affectionate nicknames on consumers’ streamer attitude. When consumers interact with human streamers, the use of affectionate nicknames leads to a more positive streamer attitude compared to when they are not used. Conversely, for virtual streamers, the absence of affectionate nicknames leads to a more favorable streamer attitude than when they are used. (2) Psychological closeness mediates the interaction between streamer type and affectionate nicknames on consumers’ streamer attitude. In the human streamer scenario, the use of affectionate nicknames enhances psychological closeness, resulting in a more positive streamer attitude. In the virtual streamer context, the absence of affectionate nicknames promotes greater psychological closeness, leading to a more favorable streamer attitude.

## 7 Discussion

### 7.1 Implications for research

This paper makes multifaceted theoretical contributions to the research field of streamer interactivity in E-commerce live streaming, while deepening and broadening its scope within the domain. Firstly, it enriches the literature on consumer responses to commercial avatars and offers a novel perspective on the effectiveness of virtual streamers as service-oriented avatars in E-commerce live streaming. This is achieved by analyzing the interactive differences between human streamers and virtual streamers, particularly their use of affectionate nicknames. Existing research primarily examines the attractiveness, credibility, and capabilities of avatars (Xu et al., 2024; He et al., 2024; Wang K. et al., 2023; Wang C. et al., 2023). In contrast, this study focuses on the consumer attitudes elicited by virtual streamers’ use of specific address forms, such as affectionate nicknames (Yao, 1991). It further highlights the risks of excessive anthropomorphism (Kim et al., 2019; Mende et al., 2019), providing empirical evidence to contextualize the debate on the anthropomorphic effects of avatars. This finding supplement research on virtual streamers in E-commerce live streaming and serves as a critical theoretical reference for designing and implementing commercial avatars.

Secondly, this study extends the role of linguistic address forms in marketing communication, particularly in the emerging field of E-commerce live streaming. By introducing psychological closeness as an intermediary mechanism, it explores the mechanisms by which address forms shape consumers’ attitudes toward human and virtual streamers. This approach offers a novel theoretical perspective for understanding consumer psychology in human–machine interactions. While prior studies have largely centered on the anthropomorphic attributes of avatars, such as sociality (Wu et al., 2023), interaction styles (Yao et al., 2024; Xu et al., 2024), and appearance (Sun and Tang, 2024), this research emphasizes the role of address forms in linguistic communication, offering new insights into human–machine interaction within E-commerce live streaming.

Lastly, this study advances the understanding of the distinctions between human-human interactions and human–machine interactions. By constructing an empirical framework, this study compares the distinct performances of human streamers and virtual streamers in linguistic communication, with a particular focus on the effects of using affectionate nicknames. This comparison provides a novel perspective for research in the field of human–machine interaction. The findings of this study reveal that, despite the increasing anthropomorphic features of avatars in terms of appearance and behavior (Liao et al., 2023; Chen H. et al., 2024; Chen T. et al., 2024; Tong et al., 2024), caution is still required when considering their impact on consumer attitudes in linguistic communication, particularly in the use of address forms (Mende et al., 2019; André et al., 2018). This discovery contributes to advancing further research on the distinctions between human-human interactions and human–machine interactions within the domain of human–machine interaction.

### 7.2 Managerial implications

The conclusions of this study offer significant insights into the practical application of service-oriented avatars within the context of E-commerce live streaming. Firstly, businesses or brands incorporating virtual streamers in real-world scenarios should exercise caution when implementing anthropomorphic strategies in linguistic communication, as the use of affectionate nicknames and other anthropomorphic address forms could potentially produce unintended effects. For example, a virtual streamer using overly abrupt and inappropriate endearments such as “babies” in an attempt to cultivate a friendly atmosphere may, in fact, provoke aversion from some viewers. Similarly, when a virtual streamer appears rigid and unnatural in explaining complex product features, it may result in comprehension difficulties and significantly detract from the viewing experience. Therefore, when virtual streamers for E-commerce live streaming, enterprises must carefully consider the limitations of current technological capabilities and their impact on consumer experience. Rather than merely transplanting the behaviors and speech of real-life streamers onto virtual counterparts, emphasis should be placed on technological innovation and contextual adaptability. By consistently refining the virtual streamer’s language processing capabilities, the naturalness of facial expressions and movements, and the rationality of interaction logic, virtual streamers can better align with consumers’ social identity expectations, thus effectively avoiding identity threats and enhancing consumer acceptance and satisfaction.

Secondly, businesses should consider enhancing the sales-assisting functions of virtual streamers to foster psychological closeness among consumers. This includes, but is not limited to, offering personalized recommendations, promptly addressing consumer inquiries, and simulating real-world shopping scenarios. For instance, when detecting a consumer’s strong interest in a particular product, the virtual streamer can proactively suggest similar items or offer styling advice. In situations where consumers display hesitation or dissatisfaction, additional discount information or solutions may be offered to alleviate their negative emotions. Furthermore, optimizing the interactive interface design—such as adopting a clear and concise layout, comfortable color schemes, and smooth animation effects—can further elevate consumers’ visual experience. Through the development and application of these functions, businesses can not only meet consumers’ diverse needs but also foster closer psychological connections during interactions, thereby promoting the development of a positive streamer attitude.

### 7.3 Limitations and future research

While this study provides valuable insights, it has several limitations. First, the study primarily examines tangible products, yet the influence of streamer type may differ depending on product categories. For example, virtual streamers might be perceived as more suitable for promoting digital products or search goods (Lee et al., 2015). Future research could explore how product categorization affects the outcomes observed in this study.

Second, the dependent variable in this study focuses on consumers’ attitudes toward streamers. However, in E-commerce contexts, other metrics may better capture streamer performance. Future studies could examine alternative dependent variables to provide a more comprehensive understanding of streamer effectiveness.

Third, given their complexity, streamer attitudes and psychological closeness are rich and multidimensional constructs. This study employs a simplified questionnaire design, which might not fully capture their intricacy. Future research could incorporate additional dimensions, such as cognitive evaluations, emotional experiences, and behavioral tendencies, to better portray these constructs’ multidimensionality, leading to richer and deeper findings.

Moreover, this study primarily investigates the effects of streamer type and affectionate nicknames within a specific cultural context. Cross-cultural comparative studies could analyze how cultural values, social norms, and communication habits influence consumers’ attitudes toward streamers and affectionate nicknames. Collecting data across diverse cultural contexts would offer valuable insights into the universality or specificity of these findings and support the development of culturally adaptive E-commerce strategies.

Finally, in terms of methodology, while this study aims to simulate real-world scenarios, its external validity is limited compared to field experiments or real-world observations. Future research could collaborate with E-commerce live streaming companies or brands to conduct field experiments. Alternatively, with appropriate measures to ensure user privacy, researchers could analyze real-world behavioral data to validate and extend the findings presented in this paper.

## Data Availability

The raw data supporting the conclusions of this article will be made available by the authors, without undue reservation.

## Footnotes

^1^ www.credamo.com
